# Supplementary material for: Determination of Phloridzin and Other Phenolic Compounds in Apple Tree Leaves, Bark, and Buds Using Liquid Chromatography with Multilayered Column Technology and Evaluation of the Total Antioxidant Activity
Source: Pharmaceuticals (Basel). 2022 Feb 18;15(2):244. doi: 10.3390/ph15020244 (PMC8880626; doi:10.3390/ph15020244)
Supplement: Supplementary file 1 [file pharmaceuticals-15-00244-s001.zip › pharmaceuticals-1551405-supplementary.pdf]

Determination of phloridzin and other phenolic compounds in apple tree leaves, bark, and buds using liquid chromatography with multilayered column technology and evaluation of the total antioxidant activity.

Anežka Adamcová[1], Aleš Horna[2, 1], Dalibor Šatínský[1]*

[1] Department of Analytical Chemistry, Faculty of Pharmacy in Hradec Králové, Charles University, Czech Republic

[2] Institute of Nutrition and Diagnostics Pardubice, Sakařova 1400, 530 03 Pardubice, Czech Republic

* Corresponding author: satinsky@faf.cuni.cz, tel. +420495067228

Supplementary material

1. The list of published HPLC conditions for determination of different phenolic substances in apple leaves 2

2. HPLC method 2

2.1. Chromatograms of mixed standard solution in tested stationary phases determined at wavelength of 254 nm 2

2.1.1. YMC Triart C18 (100 × 4.6 mm, 5 µm particle size) 2

2.1.2. YMC Triart PFP plus (150 × 4.6 mm, 5 µm particle size) 3

2.1.3. Discovery® HS C18 (150 × 4.6 mm, 5 µm particle size) 3

2.1.4. Kinetex® F5 100A (150 × 4.6 mm, 2.6 µm particle size) 4

2.1.5. *Kinetex® C18 100A (150 × 4.6 mm, 2.6 µm particle size)* 4

2.1.6. Luna Omega Polar C18 (150 × 4.6 mm, 5 µm particle size) 5

2.1.7. *Kinetex 100A Biphenyl (150 × 4.6 mm, 5 µm particle size)* 5

2.1.8. Ascentis Express RP amide (150 × 4.6 mm, 2.7 µm particle size) 6

2.2. The parameters of chromatography separation of tested columns 7

2.3. Optimization of extraction procedure – the effect of pH 8

3. Flow analysis with CoulArray Detector 8

3.1. FIAgram 8

3.2. *The total antioxidant activity of tested cultivars* 9

4. Graphical evaluation of concentration of individual phenolic compounds 9

1. The list of published HPLC conditions for determination of different phenolic substances in apple leaves

Table S1. List of published HPLC conditions for determination of different phenolic substances in apple leaves.

Sample	Analytes	Column	Mobile phase	Det.	Ref.
Apple leaves	avicularin, quercetin-3-galactoside, caffeic acid, catechin, epicatechin, chlorogenic acid, isoquercitrin, phloretin, phloridzin, quercitrin, rutin	YMC-Pack ODS-A C18 (250 × 4.6mm, 5µm)<br><br>Precolumn: YMC-Triart C18 (10 × 3 mm, 5 µm)	A: ACN<br>B: 2% $CH_3COOH + H_2O$	PDA	[1]
Apple leaves	chlorogenic acid, isoquercitrin, quercitrin, p-hydroxybenzoic acid, quercetin-3-galactoside, phloridzin, rutin	Thermo Scientific Aquasil C18 (250 × 4.6 mm, 5 µm)	A: ACN<br>B: 1 mM phosphoric acid	PDA	[2]
Apple leaves	epikatechin, isoquercitrin, quercitrin, phloretin phloridzin,	Synergi MAX RP80, C_{12} (250 × 4.6, 4 µm)	A: ACN<br>B: 0.01% trifluoroacetic acid	PDA	[3]

2. HPLC method

2.1. Chromatograms of mixed standard solution in tested stationary phases determined at wavelength of 254 nm

2.1.1. YMC Triart C18 (100 × 4.6 mm, 5 µm particle size)

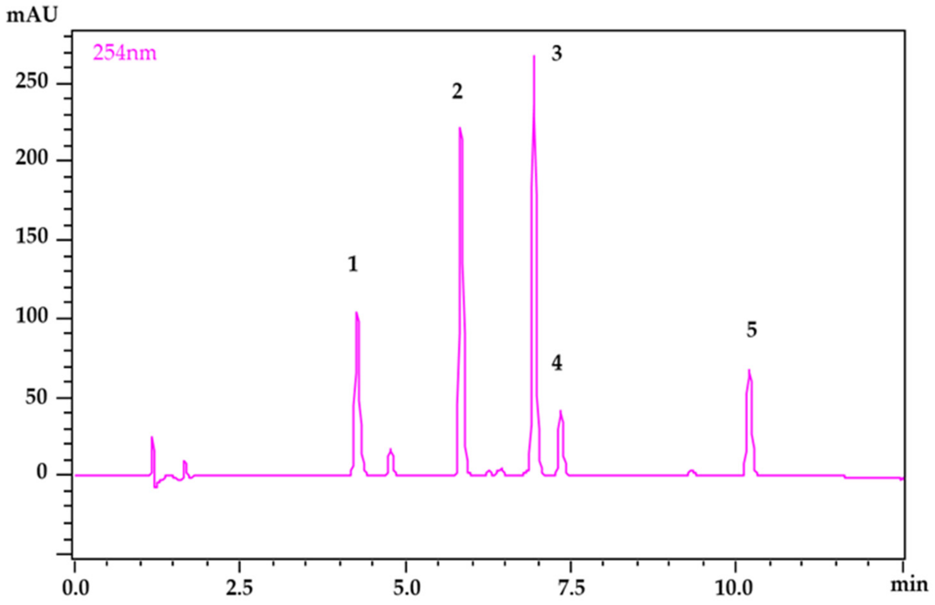

Figure S1. Chromatogram of mixed standard solution. (1) chlorogenic acid, (2) rutin, (3) quercitrin, (4) phloridzin, (5) phloretin.

2.1.2. YMC Triart PFP plus (150 × 4.6 mm, 5 µm particle size)

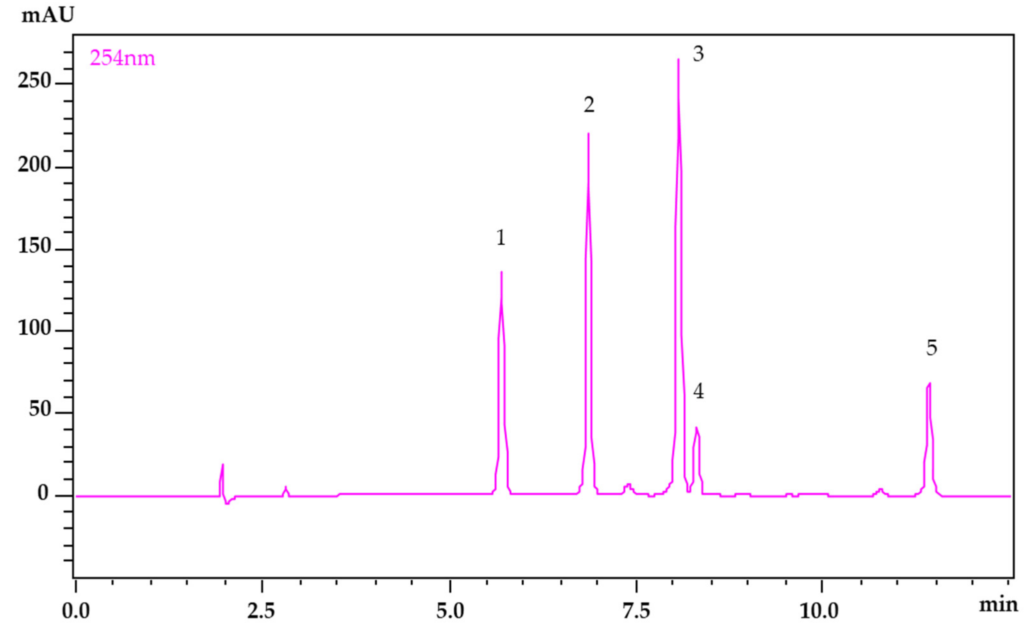

Figure S2. Chromatogram of mixed standard solution. (1) chlorogenic acid, (2) rutin, (3) quercitrin, (4) phloridzin, (5) phloretin.

2.1.3. Discovery® HS C18 (150 × 4.6 mm, 5 µm particle size)

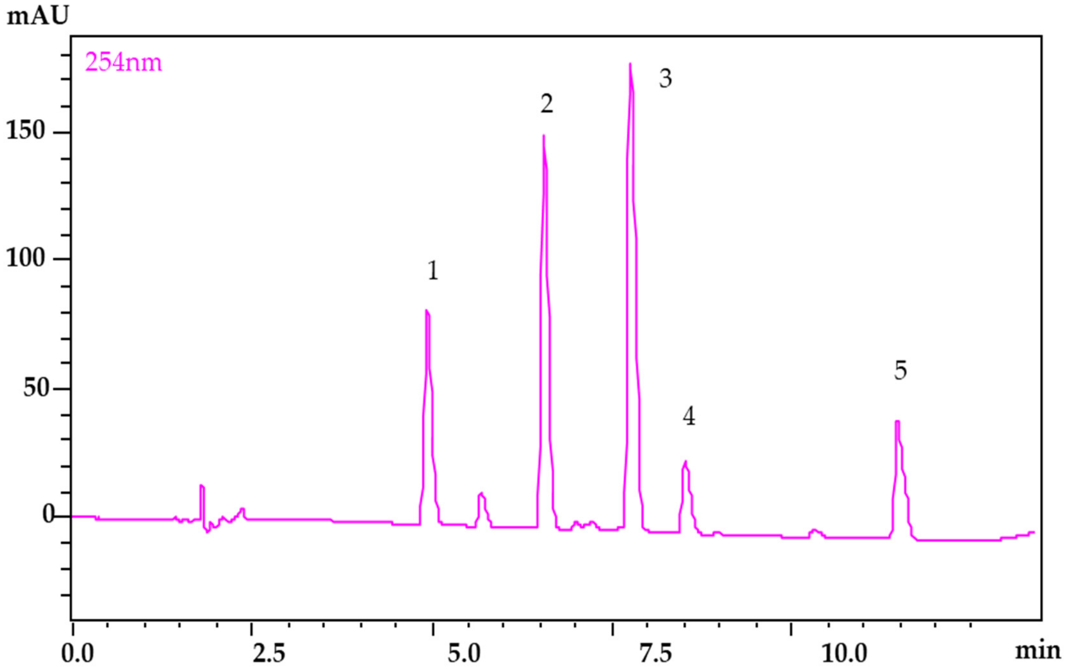

Figure S3. Chromatogram of mixed standard solution. (1) chlorogenic acid, (2) rutin, (3) quercitrin, (4) phloridzin, (5) phloretin.

2.1.4. *Kinetex® F5 100A (150 × 4.6 mm, 2.6 µm particle size)*

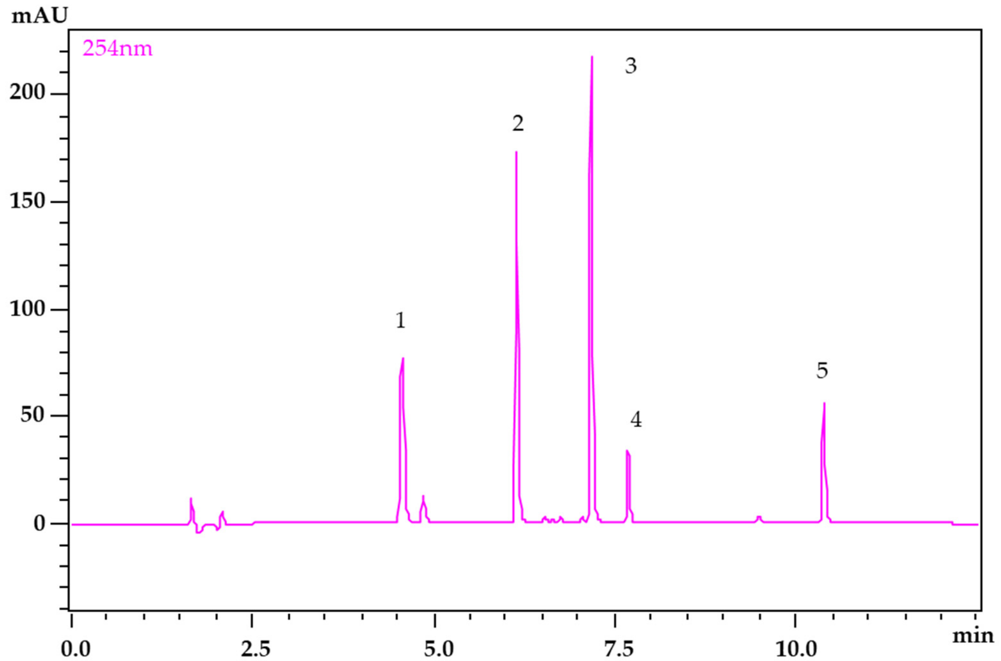

Figure S4. Chromatogram of mixed standard solution. (1) chlorogenic acid, (2) rutin, (3) quercitrin, (4) phloridzin, (5) phloretin.

2.1.5. *Kinetex® C18 100A (150 × 4.6 mm, 2.6 µm particle size*)

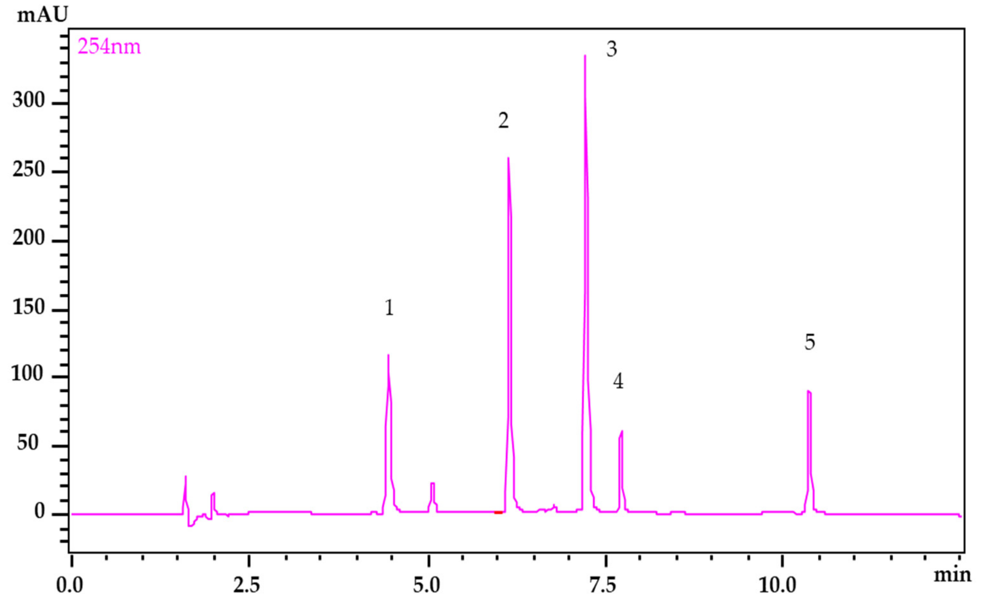

Figure S5. Chromatogram of mixed standard solution. (1) chlorogenic acid, (2) rutin, (3) quercitrin, (4) phloridzin, (5) phloretin.

2.1.6. *Luna Omega Polar C18 (150 × 4.6 mm, 5 µm particle size)*

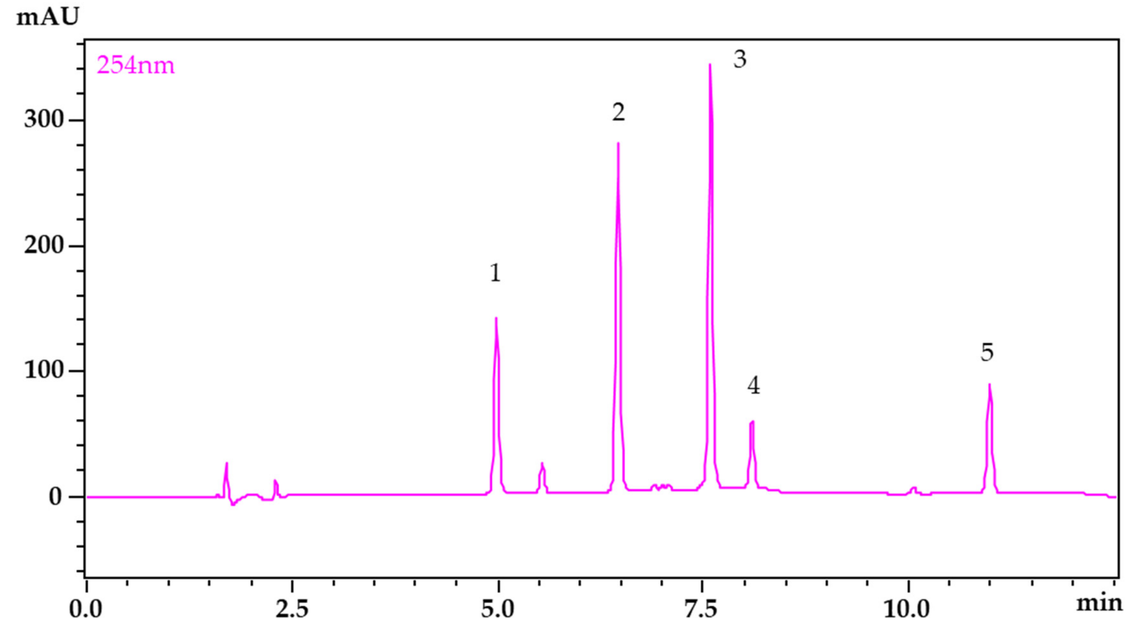

Figure S6. Chromatogram of mixed standard solution. (1) chlorogenic acid, (2) rutin, (3) quercitrin, (4) phloridzin, (5) phloretin.

2.1.7. *Kinetex 100A Biphenyl (150 × 4.6 mm, 5 µm particle size*)

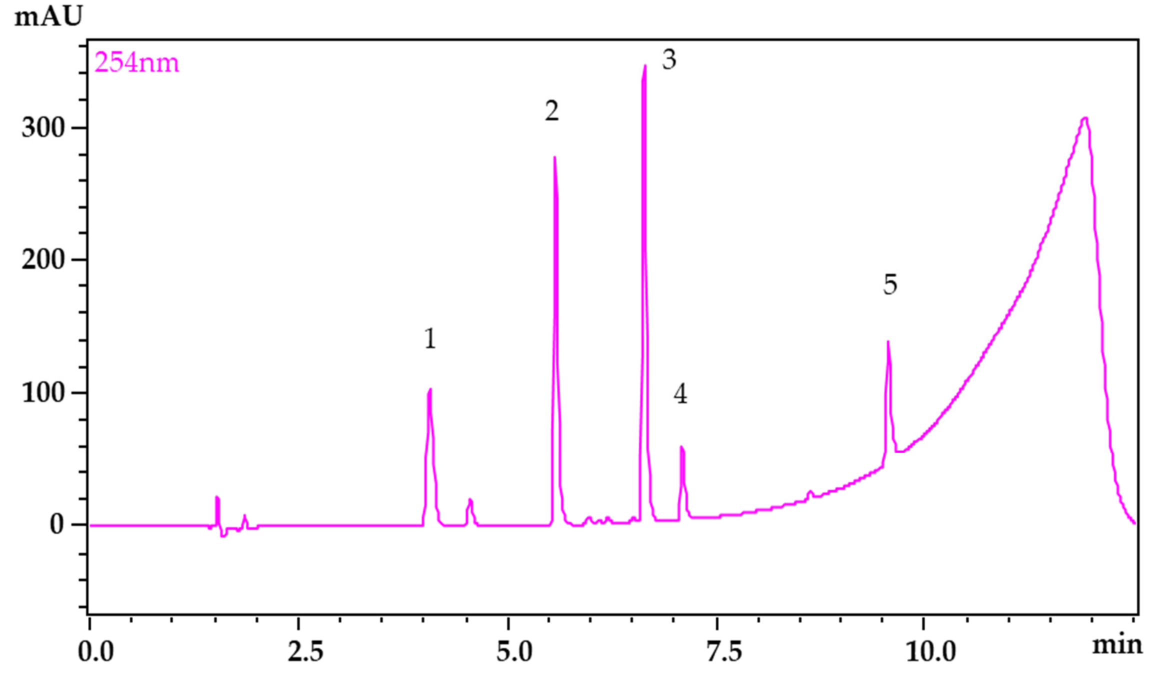

Figure S7. Chromatogram of mixed standard solution. (1) chlorogenic acid, (2) rutin, (3) quercitrin, (4) phloridzin, (5) phloretin.

2.1.8. Ascentis Express RP amide (150 × 4.6 mm, 2.7 µm particle size)

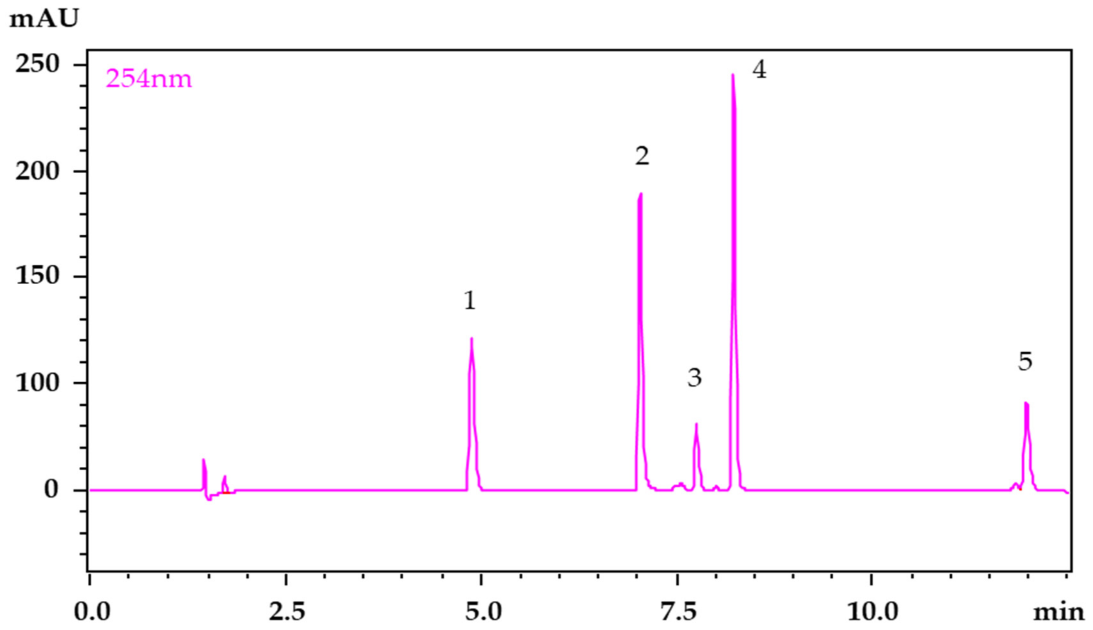

Figure S8. Chromatogram of mixed standard solution. (1) chlorogenic acid, (2) rutin, (3) phloridzin, (4) quercitrin, (5) phloretin.

2.2. The parameters of chromatography separation of tested columns

Table S2: The parameters of chromatography separation of phenolic compounds on tested columns

Column	t_R[a] (min)	A_R[b]	R_S[c]	$W_{h(5\%)}$[d]	$W_{h(50\%)}$[e]	P_c[f]	p[g] [MPa]
**YMC Triart C18 100 × 4.6 mm; 5 µm, 12 nm**							6.5
Chlorogenic acid	4.26	1.22	22.43	0.17	0.08	24	
Rutin	5.83	1.25	11.11	0.15	0.07	34	
Quercitrin	6.94	1.23	8.10	0.15	0.07	34	
Phloridzin	7.34	1.23	2.90	0.15	0.07	42	
Phloretin	10.18	1.20	18.99	0.18	0.08	26	
**YMC Triart C18 ExRS 150 × 4.6 mm; 5 µm, 8 nm**							9.5
Chlorogenic acid	4.35	1.15	21.20	0.16	0.07	31	
Rutin	5.76	1.25	10.58	0.14	0.06	38	
Quercitrin	6.89	1.23	8.82	0.14	0.07	37	
Phloridzin	7.45	1.19	4.34	0.14	0.07	40	
Phloretin	10.38	1.19	22.23	0.15	0.07	37	
**YMC Triart PFP plus 150 × 4.6 mm; 5 um, 12 nm**							6.7
Chlorogenic acid	5.66	1.12	26.30	0.16	0.07	28	
Rutin	6.83	1.10	8.46	0.16	0.07	29	
Quercitrin	8.03	1.09	8.76	0.17	0.07	46	
Phloridzin	8.26	1.13	1.68	0.15	0.07	47	
Phloretin	11.32	1.10	20.82	0.20	0.08	30	
**Discovery HS C18 150 × 4.6 mm, 5 µm**							7.7
Chlorogenic acid	4.63	1.41	16.21	0.22	0.11	27	
Rutin	6.14	1.52	7.61	0.20	0.11	34	
Quercitrin	7.26	1.61	5.49	0.22	0.12	33	
Phloridzin	7.97	1.65	3.32	0.22	0.18	35	
Phloretin	10.73	1.76	11.81	0.26	0.14	30	
**Kinetex F5 100A 150 × 4.6 mm, 2.6 µm**							22
Chlorogenic acid	4.56	1.34	32.77	0.13	0.06	43	
Rutin	6.14	1.55	11.67	0.10	0.04	43	
Quercitrin	7.17	1.48	8.37	0.10	0.04	37	
Phloridzin	7.68	1.45	5.79	0.10	0.04	57	
Phloretin	10.38	1.35	29.17	0.11	0.05	44	
**Kinetex C18 100A 150 × 4.6 mm, 2.6 µm**							21.5
Chlorogenic acid	4.45	1.47	16.11	0.16	0.07	32	
Rutin	4.45	1.47	16.11	0.13	0.07	32	
Quercitrin	7.23	1.64	10.35	0.13	0.05	39	
Phloridzin	7.73	1.42	5.01	0.11	0.05	53	
Phloretin	10.37	1.42	26.32	0.13	0.05	53	
**Luna Omega Polar C18 150 × 4.6 mm, 5 µm**							6.7
Chlorogenic acid	4.98	1.17	27.95	0.14	0.06	37	
Rutin	6.46	1.19	12.78	0.13	0.05	37	
Quercitrin	7.59	1.15	10.09	0.13	0.06	32	
Phloridzin	8.09	1.15	4.34	0.14	0.06	57	
Phloretin	10.98	1.08	23.37	0.15	0.07	34	
**Kinetex 100A Biphenyl 150 × 4.6 mm, 5 µm**							9
Chlorogenic acid	4.07	1.24	19.6	0.18	0.09	43	
Rutin	5.57	1.53	11.36	0.13	0.05	56	
Quercitrin	6.63	1.55	9.68	0.13	0.05	44	
Phloridzin	7.10	1.54	4.27	0.13	0.05	58	
Phloretin	9.56	1.47	21.62	0.14	0.06	51	
**Ascentis Express RP Amide 150 × 4.6 mm, 2.7 µm**							19.6
Chlorogenic acid	4.88	1.37	29.28	0.16	0.07	43	
Rutin	7.03	1.59	16.98	0.13	0.05	36	
Phloridzin	7.76	1.39	6.99	0.11	0.05	58	
Quercitrin	8.23	1.40	4.65	0.12	0.05	51	
Phloretin	11.98	1.28	30.47	0.16	0.07	55	

[a] *Retention time*
[b] *Peak symmetry*
[c] *Peak resolution*
[d] *Peak width at 5% of the peak height*
[e] *Peak width at 50% of the peak height*
[f] *Peak capacity (counting with time of gradient elution, 12.51 minutes)*
[g] *Pressure*

2.3. Optimization of extraction procedure – the effect of pH

Table S3: Content of phenolic compounds in leaves of 9 cultivars (all values in mg/g of dried weight (DW)).

Cultivar	Type of extraction solvent (mg/g±SD)	
	**Methanol + 0.1% formic acid (pH = 2.75)**	**Methanol + 0.1% acetic acid (pH = 3.25)**
´Melrose´	50.06±2.21	48.04±1.19
´Melodie´	72.25±4.73	64.14±4.52
´Rubinola´	48.15±3.83	47.31±2.60
´Průsvitné letní´	58.38±1.97	56.04±2.85
´Fragrance´	54.88±4.18	46.42±18.88
´Goldstar´	49.11±1.69	44.05±2.53
´Topaz´	61.18±2.08	47.35±7.92
´Bohemia Gold´	61.44±1.86	64.20±4.81
´Gloster´	53.48±2.11	53.62±0.93

Concentrations ± standard deviation (RSD, %) calculated from the mean of 3 measurements.

3. Flow analysis with CoulArray Detector

3.1. FIAgram

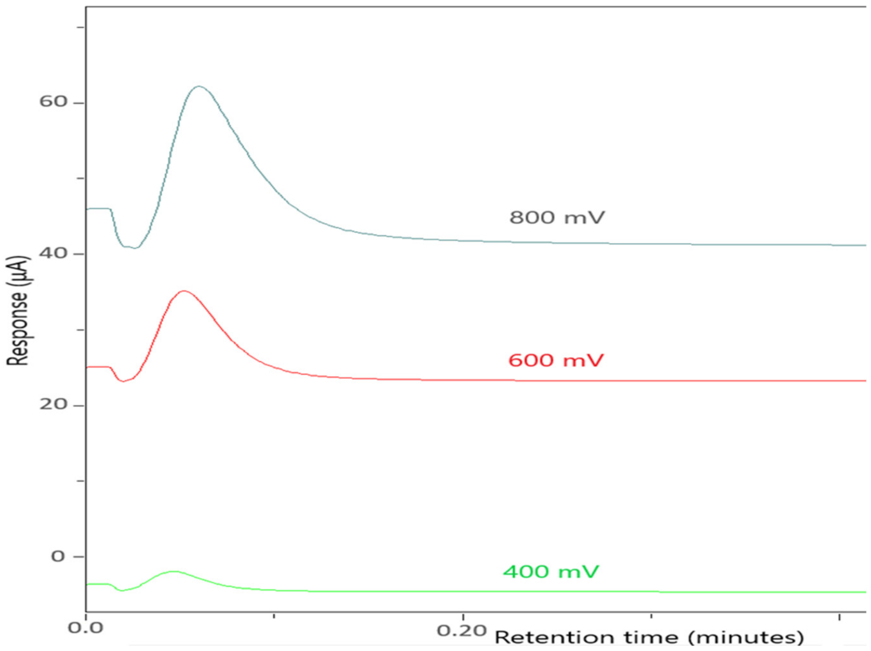

Figure S9. FIAgram of apple bark extract.

3.2. *The total antioxidant activity of tested cultivars*

Table S4: The total antioxidant activity of tested cultivars in µC

Cultivar	Leaves (µC±SD)	Bark (µC±SD)	Buds (µC±SD)
´Melrose´	108.25±2.32	-	59.90±7.19
´Melodie´	92.88±2.28	69.84±0.62	102.91±6.75
´James Grieve´	95.29±2.65	88.72±2.47	85.63±2.16
´Rubinola´	96.22±2.08	74.02±2.40	75.46±4.25
´Goldstar´	71.88±1.65	64.95±2.34	-
´Meteor´	78.03±5.26	59.17±2.56	-
´Průsvitné letní´	74.88±1.29	68.80±1.35	83.14±1.47
´Topaz´	119.63±2.76	92.78±1.73	94.14±0.30
´Red Bilt´	80.01±3.94	79.77±4.80	83.88±10.42
´Spartan´	97.76±1.48	96.15±1.46	-
´Fragrance´	71.52±3.19	87.15±0.47	68.52±8.60
´Gloster´	110.10±1.06	79.63±4.80	80.90±6.30
´Bohemia Gold´	90.99±2.28	71.64±1.77	80.88±8.70

Concentrations ± standard deviation (RSD, %) calculated from the mean of 3 measurements.

4. Graphical evaluation of the concentration of individual phenolic compounds

4.1. The concentration of individual phenolic compounds expressed in mg/g DW obtained from apple leaves

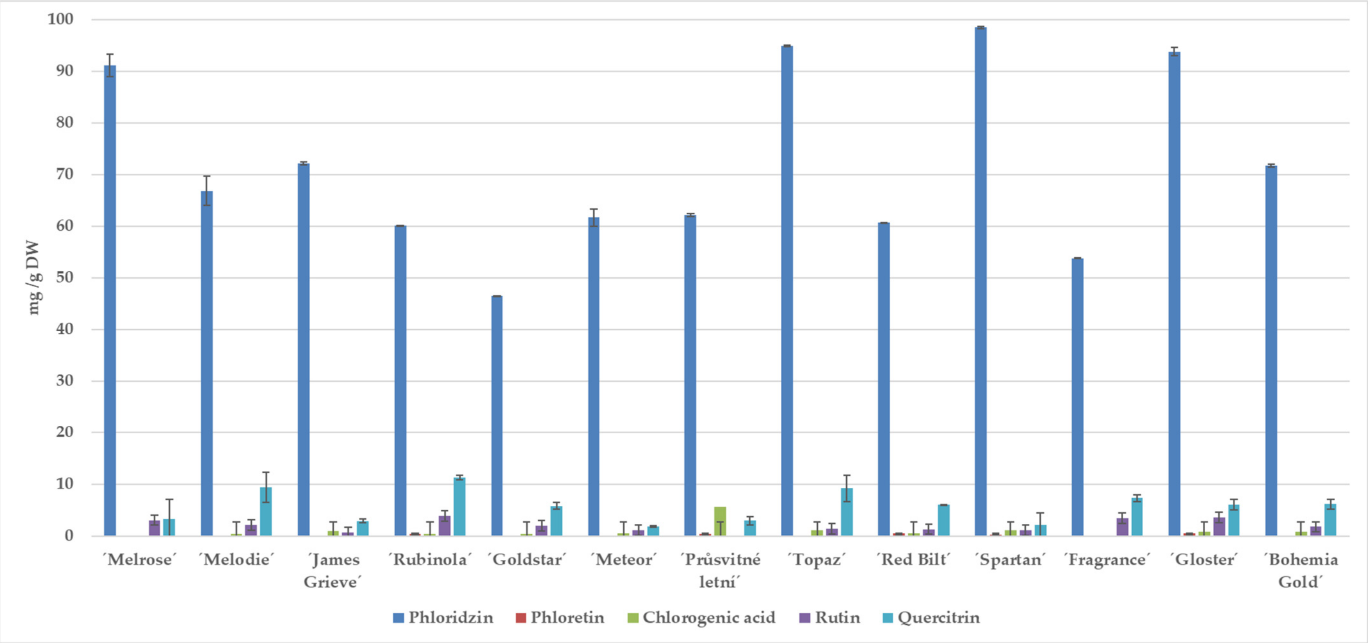

Figure S10. *The concentration of individual phenolic compounds expressed in mg/g DW obtained from apple leaves. Concentrations ± standard deviation (RSD, %) calculated from the mean of 3 measurements.*

4.2. The concentration of individual phenolic compounds expressed in mg/g DW obtained from apple bark

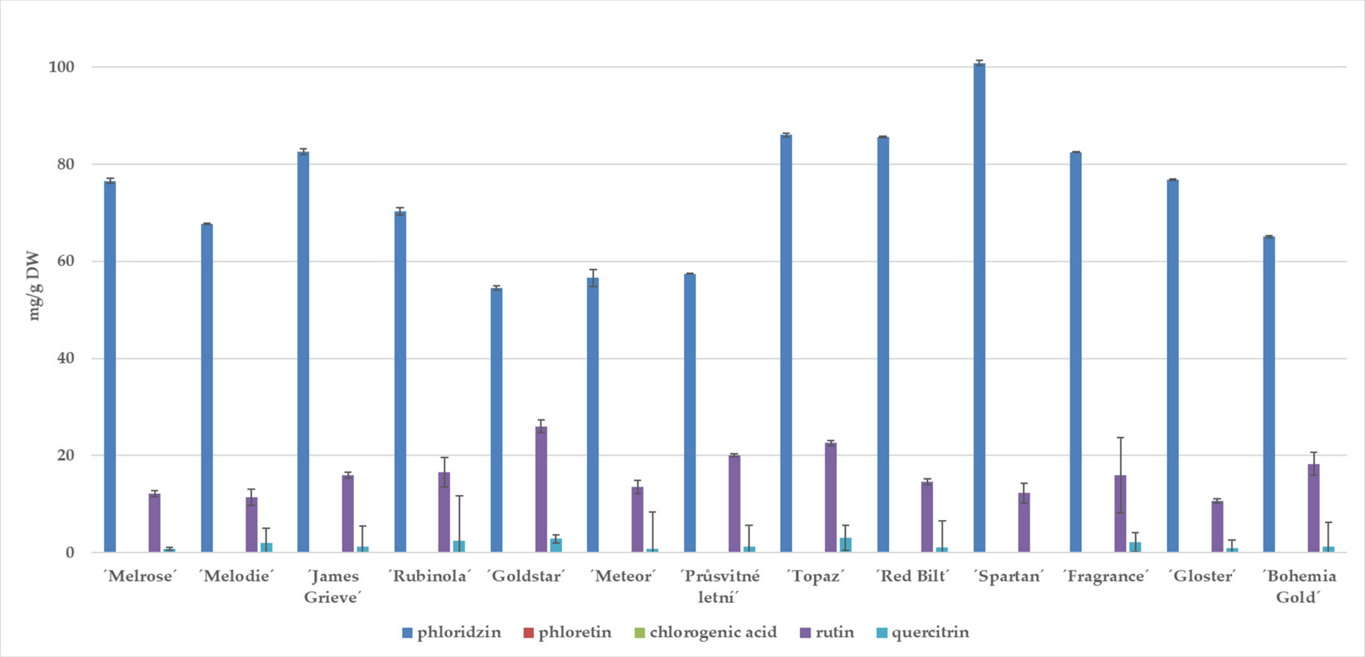

Figure S11. *The concentration of individual phenolic compounds expressed in mg/g DW obtained from apple bark. Concentrations ± standard deviation (RSD, %) calculated from the mean of 3 measurements.*

4.3. The concentration of individual phenolic compounds expressed in mg/g DW obtained from apple buds

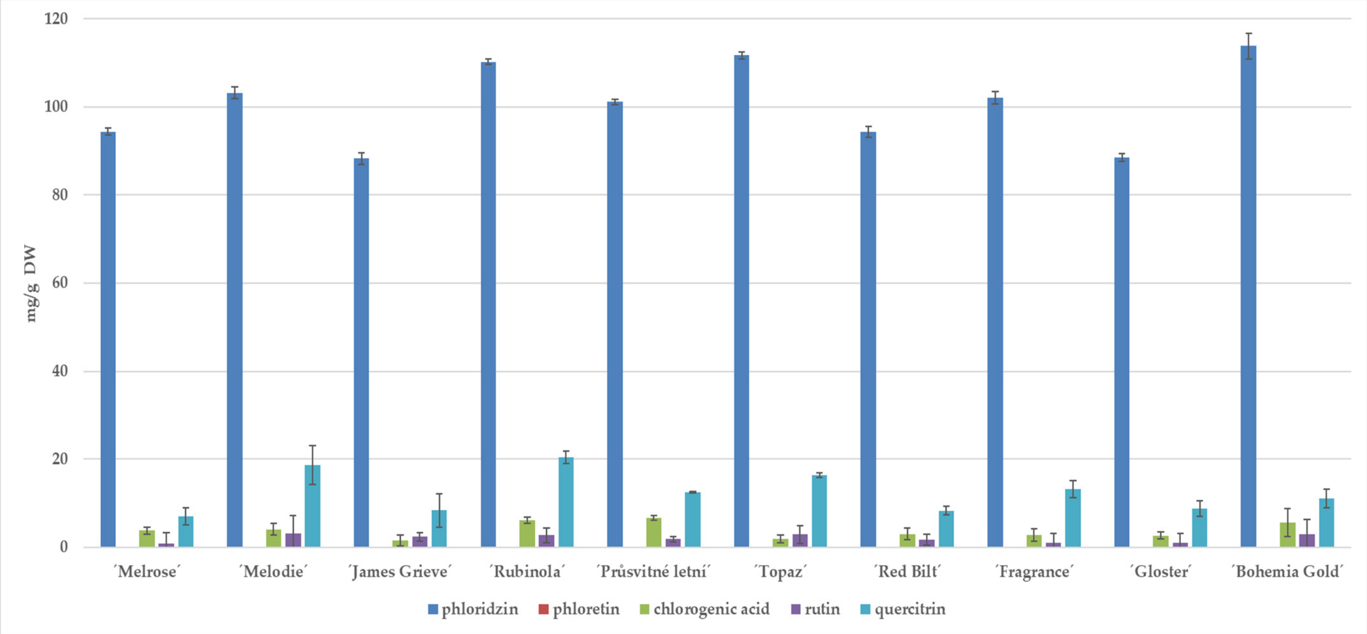

Figure S12. *The concentration of individual phenolic compounds expressed in mg/g DW obtained from apple buds. Concentrations ± standard deviation (RSD, %) calculated from the mean of 3 measurements.*

References

1. Liaudanskas, M., Viškelis, P., Raudonis, R., Kviklys, D., Uselis, N., Janulis, V. Phenolic composition and antioxidant activity of Malus domestica leaves. *Sci World J* **2014**, 306217 https://doi.org/10.1155/2014/306217

2. Sowa, A.; Zgórka, G.; Szykuła, A.; Franiczek, R.; Żbikowska, B.; Gamian, A.; Sroka, Z. Analysis of Polyphenolic Compounds in Extracts from Leaves of Some Malus domestica Cultivars: Antiradical and Antimicrobial Analysis of These Extracts. *BioMed Res Int* **2016**, *2016*, 1-12 https://doi.org/10.1155/2016/6705431

3. Rana, S.; Kumar, S.; Rana, A.; Sharma, V.; Katoch, P.; Padwad, Y.; Bhushan, S. Phenolic constituents from apple tree leaves and their in vitro biological activity. *Ind Crop Prod* **2016**, *90*, 118-125. https://doi.org/10.1016/j.indcrop.2016.06.027
